# Full-length transcriptome analysis and identification of transcript structures in *Eimeria necatrix* from different developmental stages by single-molecule real-time sequencing

**DOI:** 10.1186/s13071-021-05015-7

**Published:** 2021-09-27

**Authors:** Yang Gao, Zeyang Suding, Lele Wang, Dandan Liu, Shijie Su, Jinjun Xu, Junjie Hu, Jianping Tao

**Affiliations:** 1 College of Veterinary Medicine, Yangzhou University, Yangzhou, 225009 China; 2 Jiangsu Co-Innovation Center for Prevention and Control of Important Animal Infectious Diseases and Zoonoses, Yangzhou University, Yangzhou, 225009 China; 3 Jiangsu Key Laboratory of Zoonosis, Yangzhou University, Yangzhou, 225009 China; 4 Biology Department, Yunnan University, Kunming, 650500 China

**Keywords:** *Eimeria necatrix*, Novel genes, Alternative splicing, Alternative polyadenylation, Long non-coding RNAs, Fusion transcripts, Transcription factors

## Abstract

**Background:**

*Eimeria necatrix* is one of the most pathogenic parasites, causing high mortality in chickens. Although its genome sequence has been published, the sequences and complete structures of its mRNA transcripts remain unclear, limiting exploration of novel biomarkers, drug targets and genetic functions in *E. necatrix*.

**Methods:**

Second-generation merozoites (MZ-2) of *E. necatrix* were collected using Percoll density gradients, and high-quality RNA was extracted from them. Single-molecule real-time (SMRT) sequencing and Illumina sequencing were combined to generate the transcripts of MZ-2. Combined with the SMRT sequencing data of sporozoites (SZ) collected in our previous study, the transcriptome and transcript structures of *E. necatrix* were studied.

**Results:**

SMRT sequencing yielded 21,923 consensus isoforms in MZ-2. A total of 17,151 novel isoforms of known genes and 3918 isoforms of novel genes were successfully identified. We also identified 2752 (SZ) and 3255 (MZ-2) alternative splicing (AS) events, 1705 (SZ) and 1874 (MZ-2) genes with alternative polyadenylation (APA) sites, 4019 (SZ) and 2588 (MZ-2) fusion transcripts, 159 (SZ) and 84 (MZ-2) putative transcription factors (TFs) and 3581 (SZ) and 2039 (MZ-2) long non-coding RNAs (lncRNAs). To validate fusion transcripts, reverse transcription-PCR was performed on 16 candidates, with an accuracy reaching up to 87.5%. Sanger sequencing of the PCR products further confirmed the authenticity of chimeric transcripts. Comparative analysis of transcript structures revealed a total of 3710 consensus isoforms, 815 AS events, 1139 genes with APA sites, 20 putative TFs and 352 lncRNAs in both SZ and MZ-2.

**Conclusions:**

We obtained many long-read isoforms in *E. necatrix* SZ and MZ-2, from which a series of lncRNAs, AS events, APA events and fusion transcripts were identified. Information on TFs will improve understanding of transcriptional regulation, and fusion event data will greatly improve draft versions of gene models in *E. necatrix*. This information offers insights into the mechanisms governing the development of *E. necatrix* and will aid in the development of novel strategies for coccidiosis control.

**Graphical Abstract:**

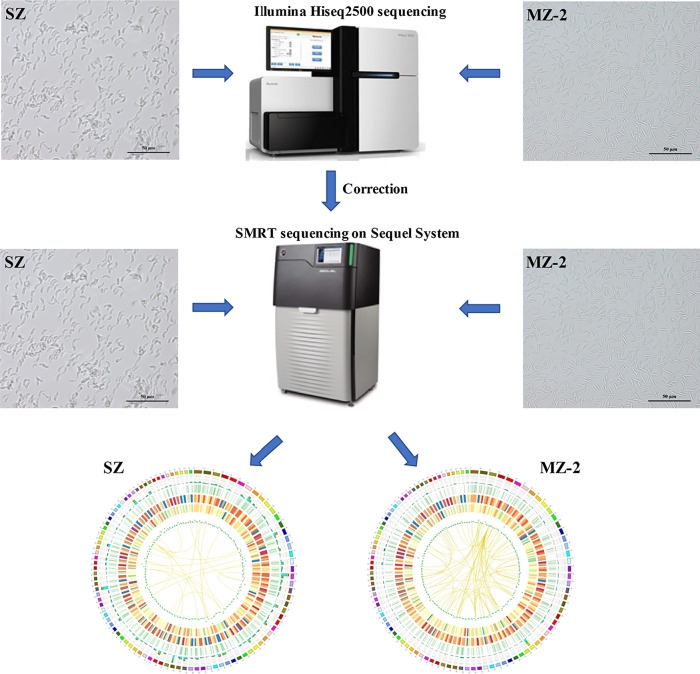

**Supplementary Information:**

The online version contains supplementary material available at 10.1186/s13071-021-05015-7.

## Background

Avian coccidiosis, caused by protozoan parasites of the genus *Eimeria*, is one of the most important diseases affecting the poultry industry [1, 2]. The economic impact of coccidiosis is over US$3 billion per annum owing to production losses combined with costs of prevention and treatment [3]. Poultry coccidiosis is currently controlled by prophylactic medication and vaccines, but the appearance of drug resistance [4] and the close attention paid to food safety [5] have made immunoprophylaxis an attractive strategy for parasite control [6]. Live vaccines, which are composed of virulent and/or attenuated strains of *Eimeria*, have significant drawbacks, such as high costs, low yield of oocysts, variation between species and the risk of introducing new species or unexpected pathogens into a flock [7]. Subunit recombinant vaccines utilizing safe antigens work by inducing incomplete immune protection [2] and have shown great promise over the last few decades [8].

*Eimeria necatrix* is a highly pathogenic pathogen that mainly colonizes the midsegments of the small intestine, causing weight loss, poor feed conversion and high mortality [9]. This parasite undergoes a complex life-cycle that includes an exogenous phase (sporogony) and an endogenous phase (schizogony and gametogony) [10]. Sporozoites (SZ) are the initial invasive stage and are characterized by a unique complex of structures specialized for the invasion of host cells [11]. Merozoites (MZ) liberated from schizonts enter adjacent epithelial cells, where they develop into next-generation schizonts or become macrogametes or microgametes. Second-generation schizonts of *E. necatrix*, which parasitize the lamina propria, with each schizont producing > 150 second-generation merozoites (MZ-2), are considered to be the most concerning stage in terms of pathogenicity due to the severe damage they cause to intestinal tissue [12, 13]. The differentiation and development of distinct biological stages of this apicomplexan are dependent on tightly regulated gene transcription [14]. Consequently, information on RNA sequences is crucial for understanding the *E. necatrix* transcriptome and evaluating the structure of genes associated with stage differentiation.

Short-read sequencing using Illumina technology (Illumina Inc., San Diego, CA, USA), referred to here as Illumina sequencing short reads, is an effective method for accurately analyzing RNA transcripts and gene expression levels [15, 16]. However, the lengths of Illumina sequencing short reads make them poorly suited for examining some biological processes, such as the assembly and determination of complex genomic regions, gene isoform detection and methylation detection. Single-molecule real-time (SMRT) sequencing has been applied to effectively capture the full-length sequences of transcripts but has limitations, such as a high error rate. However, combined PacBio SMRT sequencing [Pacific Biosciences of California, Inc. (PacBio), Menlo Park, CA, USA) and Illumina sequencing can be applied to overcome the disadvantages of each individual technique separately [17]. Combined Illumina short-read sequencing and SMRT sequencing has allowed researchers to successfully analyze the transcriptomes of *Ancylostoma ceylanicum* [18], Zebrafish (*Danio rerio*) [19] and *Medicago sativa* L. [20], resulting in novel information on alternative splicing (AS) events, fusion genes, transcription factors (TFs), long non-coding RNAs (lncRNAs) and new transcripts. Such research provides useful transcriptome information and serves as a valuable resource for further research.

Although the genome sequence of *E. necatrix* has been published [21], the sequences and completed structures of messenger RNA (mRNA) transcripts remain unclear, which limits further exploration of novel biomarkers, drug targets and genetic functions in *E. necatrix*. In the present study, we conducted a combination of SMRT sequencing and Illumina sequencing to generate the transcripts of MZ-2. Combined with the SMRT sequencing data of SZ collected in our previous study [22], we studied the transcriptome and transcript structures of *E. necatrix*. The data provided full-length sequences and gene isoforms of *E. necatrix* transcripts that will enhance our understanding of gene structure in this parasite and help reveal mechanisms governing the development of *Eimeria* parasite.

## Methods

### Animals and parasites

A total of 300 one-day-old yellow-feathered broilers were obtained from the Jiangsu Jinghai Poultry Industry Group Co., Ltd (Nantong, Jiangsu, China). Chickens were housed in *Eimeria*-free isolation cages and provided with clean water and adequate feed without anticoccidial drugs. Chicken feces were collected and analyzed by salt flotation and light microscopy to confirm the absence of oocysts in each chicken before the experimental inoculations. Chickens between 4 and 5 weeks of age were used to prepare MZ-2 of *E. necatrix*. The Yangzhou *E. necatrix* strain used in the present study was originally isolated from chickens that died from *E. necatrix* infection in 2009 (Yangzhou, Jiangsu, China). The identity of the strain was determined using the single-oocyst method described previously [23] and confirmed by microscopic examination and sequence analysis of the ribosomal RNA (rRNA) gene internal transcribed spacer region [24]. All animal experiments were approved by and conducted in strict accordance with the guidelines of the Animal Care and Use Committee of the College of Veterinary Medicine, Yangzhou University. The physical condition of the animals was monitored every day throughout the experimental period.

### Preparation of MZ-2

The preparation of MZ-2 was performed using methods described previously [25, 26]. Briefly, chickens were infected with 1.0 × 10^4^ sporulated *E. necatrix* oocysts. Infected intestinal tissues were removed at 136 h post-infection, cut longitudinally and rinsed three times with ice-cold phosphate-buffered saline (PBS; pH 7.4). The mucosa was scraped using two glass slides and put into 10 volumes of a solution containing 120 mM NaCl, 10 mM CaCl_2_, 3 mM K_2_HPO_4_, 20 mM Tris–HCl, 0.1% bovine serum albumin and 0.1% hyaluronidase, and incubated for 1 h at 37 °C in a thermostatic water bath. Large intestinal debris was removed by filtering through gauze, and small debris was removed by sequential filtration through 17- and 10-µm polymon mesh (Sefar Filtration Solution Co. Ltd., Suzhou, China). The mixture was then centrifuged at 1400* g* for 10 min and the pellet washed three times in ice-cold PBS. To remove red blood cells, the pellet was resuspended in lysis buffer (Solarbio, Beijing, China) and allowed to stand for 10 min at 4 °C. After three washes with ice-cold PBS, the resulting MZ-2 were purified by density-gradient centrifugation using the method described by Mo et al. [25]. Approximately l0^10^ merozoites were recovered from each chicken (Additional file 1: Figure S1). Purified MZ-2 were stored in liquid nitrogen for further use.

### RNA extraction

RNA extraction of *E. necatrix* MZ-2 was performed using methods described in our previous study [22]. Briefly, total RNA was extracted from each sample using Trizol™ reagent (Invitrogen™, Thermo Fisher Scientific, Waltham, MA, USA). RNA degradation and contamination were assessed using 1% agarose gels (Additional file 2: Figure S2). RNA concentrations were quantified using a Qubit® RNA Assay Kit and Qubit® 2.0 Fluorometer (Life Technologies, Thermo Fisher Scientific). RNA purity and integrity were evaluated using an Implen NanoPhotometer spectrophotometer (Implen, Westlake Village, CA, USA) and an RNA 6000 Nano Kit on a 2100 Bioanalyzer system (Agilent Technologies, Santa Clara, CA, USA) (Additional file 3: Figure S3).

### Library construction and sequencing

Library construction and sequencing were performed using methods described in our previous study [22]. For SMRT sequencing, 3 µg RNA from each of the high-quality samples was used as input material for library construction and transcriptome sequencing. An isoform sequencing (Iso-Seq) library was generated using a SMARTer™ PCR cDNA Synthesis Kit (PacBio) according to the manufacturer’s recommendations. SMRT sequencing was performed using the Pacific Bioscience Sequel System. In addition, a total of 3 μg RNA was used for short-read sequencing on the Illumina Hiseq 2500 platform (Illumina, Inc.).

### Data processing and functional annotation

Data processing was performed using methods described in our previous study [22]. Briefly, raw read data were processed using SMRTlink version 5.1 software (PacBio) with the following parameters: minLength, 200; minReadScore, 0.65. Circular consensus sequences (CCSs) were generated from subread BAM files with the following parameters: minPasses, 2; minPredictedAccuracy, 0.8. CCSs were then classified as full-length non-chimeric reads (FLNC) or non-full-length reads by identifying the presence of 5′ and 3′ primers and poly(A) signals (Additional file 4: Table S1). An additional round of error correction on the FLNC was performed using the iterative clustering for error correction algorithm to identify consensus isoforms [27]. The consensus isoforms were further polished with non-full-length reads to obtain high-quality isoforms with a post-correction accuracy > 99% using Arrow software [28]. Finally, the polished consensus isoforms were corrected using the Illumina RNA-Seq data with LoRDEC software [29]. The corrected polished consensus isoforms were aligned to the *E. necatrix* genome (https://www.ncbi.nlm.nih.gov/assembly/GCF_000499385.1/) using the GMAP software program for mapping and aligning cDNA sequences to a genome [30]. The SMRT sequencing data of SZ were collected in our previous study [22].

The corrected isoforms were functional annotated by performing searches against seven databases [22], including the NCBI non-redundant protein (NR) (https://www.ncbi.nlm.nih.gov/protein/), Swiss-Prot protein (https://www.uniprot.org/uniprot/), euKaryotic Ortholog Groups protein (KOG) (http://www.ncbi.nlm.nih.gov/KOG), Protein families (Pfam) (https://pfam.xfam.org/), NCBI non-redundant nucleotide (NT) (https://www.ncbi.nlm.nih.gov/nuccore), the Kyoto Encyclopedia of Genes and Genomes (KEGG) (http://www.genome.jp/kegg/) and the Gene Ontology (GO) (http://www.geneontology.org) databases.

### Gene structure analysis

Owing to the presence of the reference genome, gene structure analysis was performed using the TAPIS pipeline tool based on BLAST results. AS events were identified using SUPPA software [31]. SUPPA classifies AS events into one of seven different types: skipped exons (SE), mutually exclusive exons (MX), alternative 5’ and 3’ splice sites (A5/A3), retained introns (RI) and alternative first and last exons (AF/AL). This method can be used to effectively distinguish exon–intron structures and statistically analyze the number of introns at the transcriptome level. Alternative polyadenylation (APA) events and polyadenylation sites were identified using TAPIS and MEME, respectively [32]. Fusion transcripts were considered chimeric RNA made of two or more transcripts that can fuse at the RNA level via* trans*- or* cis*-splicing between neighboring genes [33]. Finally, candidate fusion transcripts were validated by at least two Illumina short reads [34].

### Identification of TFs and long non-coding RNAs

The animal TFDB 2.0 database [35] was used as the reference TF database. HMMER 3.0 software [36] was applied to identify TFs and assign genes to different families. Non-protein coding transcripts with lengths > 200 nucleotides (nt) were considered long non-coding RNAs (lncRNAs). To identify lncRNAs in the SMRT data, we employed four methods, including predictor of long non-coding RNAs and mRNAs based on an improved k-mer scheme (PLEK) [37], Coding Potential Calculator (CPC) [38], Coding-Non-Coding Index (CNCI) [39] and Pfam [40]. The default parameters recommended by the respective instructions were used, and transcripts predicted by all four methods were retained. LncRNAs were divided into four groups, namely the long intergenic non-coding RNA (lincRNA), antisense, sense intronic and sense overlapping groups, based on the method reported by Harrow [41].

### Reverse transcription-PCR validation of fusion transcripts

For PCR validation of fusion transcripts, gene-specific primers were designed using Primer Premier software version 5.0 (PREMIER Biosoft International, Palo Alto, CA, USA). All primers used for reverse transcription (RT)-PCR analysis are listed in Additional file 5: Table S2. The PCR products were confirmed by Sanger sequencing to ensure the authenticity of the chimeric transcripts.

## Results

### Transcriptome sequencing using SMRT

SMRT sequencing yielded 6,756,870 subreads in MZ-2, of which 322,342 were FLNC reads (Additional file 4: Table S1). The mean length of subreads was 1878 bp for MZ-2 (Additional file 6: Figure S4a). The average length of the FLNC reads was 2427 bp for MZ-2 (Additional file 6: Figure S4b). All polished consensus reads were corrected using the approximately 70 million Illumina cleaned reads as input data (Additional file 7: Table S3), and a total of 192,045 corrected polished consensus reads were obtained from the MZ-2 library (Additional file 8: Table S4). The average length of polished consensus isoforms was 2368 bp (N50 = 2780 bp) from the MZ-2 library (Additional file 6: Figure S4c). After subsequent assembly, a total of 4007 genes were identified from the MZ-2 library (Additional file 4: Table S1).

### Genome mapping

**Fig. 1:**
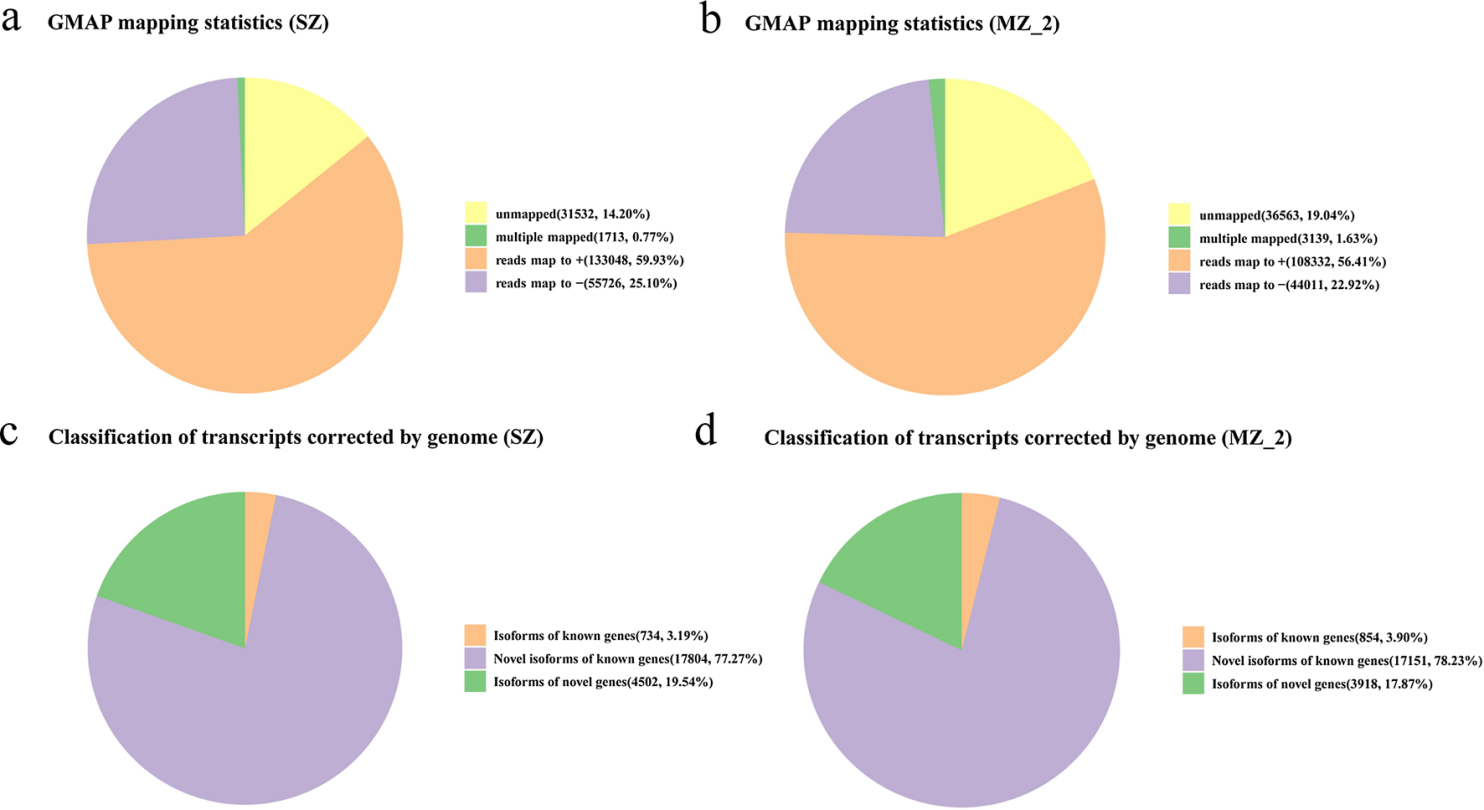
GAMP analysis and classification of corrected transcripts. **a**, **b** GAMP analysis of corrected reads in the reference genomes of SZ and MZ-2, respectively. **c**, **d **Classification of corrected transcripts mapped to the reference genomes of SZ and MZ-2, respectively. Abbreviations: SZ, sporozoites; MZ-2, second-generation merozoites

A total of 190,487 and 155,482 reads were mapped to the reference genome from the SZ and MZ-2 libraries, respectively. These reads could be divided into five groups. The unmapped group consisted of 31,532 (SZ, 14.2%) and 36,563 (MZ-2, 19.04%) reads with no significant mapping to the draft genome. The multiple mapped group contained 1713 (SZ, 0.77%) and 3139 (MZ-2, 1.63%) reads showing multiple alignments. The uniquely mapped group comprised 188,774 (SZ, 85.03%) and 152,343 (MZ-2, 79.33%) reads mapped to one unique location in the genome. Those mapped to the ‘+’ group included 133,048 (SZ, 59.93%) and 108,332 (MZ-2, 56.41%) reads mapped to the positive strand of the genome; those mapped to the ‘−’ group included 55,726 (SZ, 25.1%) and 44,011 (MZ-2, 22.92%) reads mapped to the opposite strand of the genome (Fig. 1a, b; Additional file 9: Table S5).

### Novel genes and transcript findings

**Fig. 2:**
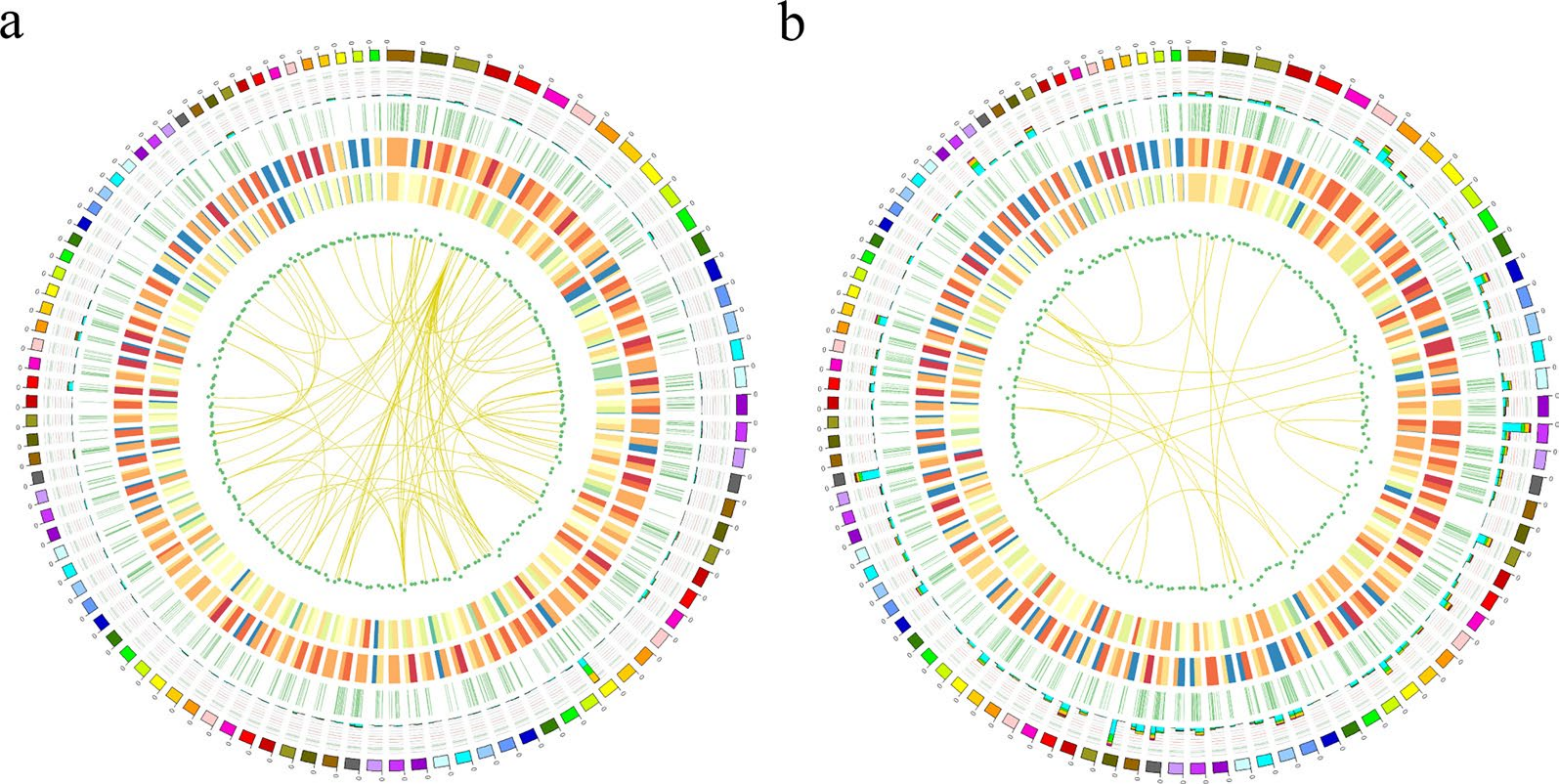
Visualization of SMRT sequence data at the genome-wide level using the Circos software package. **a**, **b** Circos visualization of SMRT sequence data at the genome-wide level in SZ and MZ-2, respectively. From outside to inside, the Circos shows chromosomes of *Eimeria necatrix*, AS sites, APA sites, the distribution of novel transcript densities (higher densities are a deeper red), the distribution of novel gene densities (higher densities are a deeper red) and the distribution of lncRNA densities (lower densities are closer to the center). Linkage of fusion transcripts is shown in purple (intra-chromosomal) and yellow (inter-chromosomal). Abbreviations: AS, Alternative splice; APA, alternative polyadenylation; IncRNA, long non-coding RNA

All polished consensus isoforms were compared against the genome sequence using GMAP, and 23,040 and 21,923 isoforms were mapped to the reference genome for SZ and MZ-2, respectively. Mapped isoforms were divided into three types: isoforms of known genes (SZ, 734; MZ-2, 854), novel isoforms of known genes (SZ, 17,804; MZ-2, 17,151) and isoforms of novel genes (SZ, 4502; MZ-2, 3918) (Figs. 1c, d, 2a, b).

### Functional annotation of novel genes

**Fig. 3:**
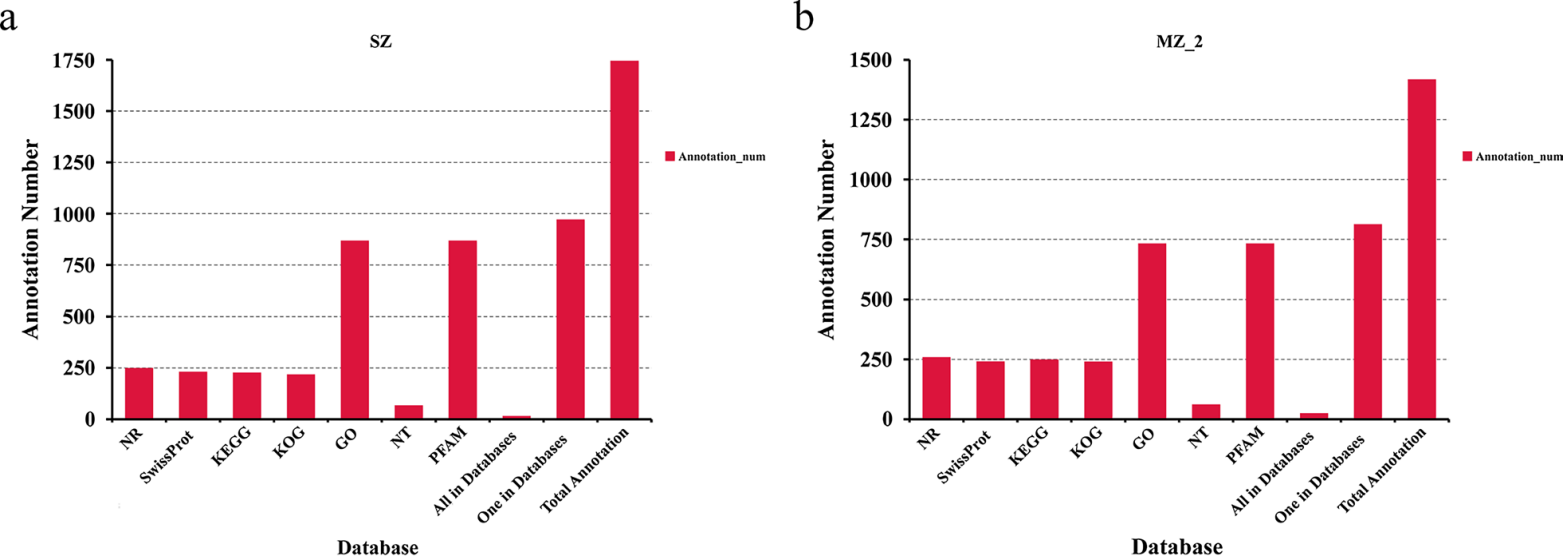
Functional annotation of corrected transcripts. **a**, **b** Functional annotation of SZ and MZ-2 transcripts using seven databases (GO, KEGG, KOG, NR, NT, PFAM, Swiss-Prot). Abbreviations: GO, Gene Ontology; KEGG, Kyoto Encyclopedia of Genes and Genomes; KOG, Cluster of EuKaryotic Orthologous Groups of Proteins; NR, Non-Redundant Protein Database; NT, Non-Redundant Nucleotide Database; PFAM, database of protein families; Swiss-Prot, protein sequence database

A total of 1743 and 1416 novel genes were successfully annotated from SZ and MZ-2, respectively, using the NR, NT, Swiss-Prot, GO, KOG, Pfam and KEGG databases (Fig. 3a, b). Over half of the novel genes (SZ,* N* = 969, 55.59%; MZ-2,* N* = 811, 57.27%) were annotated by at least one database.

**Fig. 4:**
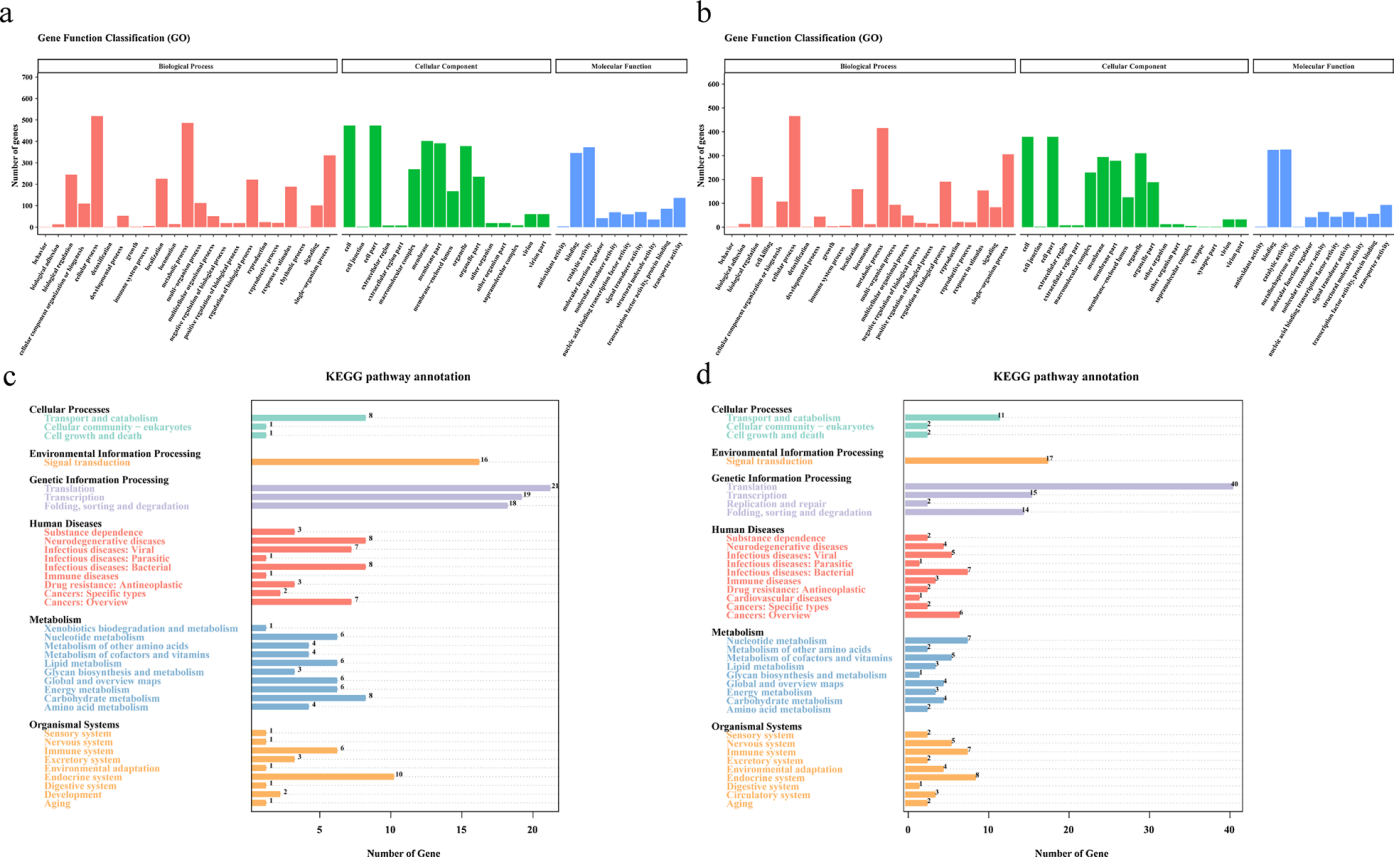
Gene functional classification and KEGG pathway annotation. **a**, **b** Distribution of GO terms of biological processes, cellular components and molecular functions for all annotated transcripts in SZ (**a**) and MZ-2 (**b**). **c**, **d** KEGG pathways enriched with transcripts in SZ (**c**) and MZ-2 (**d**)

GO analysis showed that 866 novel genes in SZ were clustered into 49 GO terms, including 16 cellular components, 10 molecular functions and 23 biological processes. The annotated genes were mainly involved in functions of cellular processes, metabolic processes, cell, cell parts and membrane (Fig. 4a). A total of 730 novel genes in MZ-2 were clustered into 52 GO terms, including 18 cellular components, 11 molecular functions and 23 biological processes. The annotated genes were mainly involved in functions of cellular processes, metabolic processes, cell, cell part and binding (Fig. 4b).

Further validating the molecular functions and biological pathways, KEGG analysis showed that 225 novel genes in SZ were mapped onto 115 KEGG pathways, among which the overrepresented pathways included translation, transcription, folding, sorting and degradation (Fig. 4c). Similarly, 247 novel genes in MZ-2 were mapped onto 128 KEGG pathways, among which the overrepresented pathways included translation, signal transduction and transcription (Fig. 4d).

### AS event analysis

**Fig. 5:**
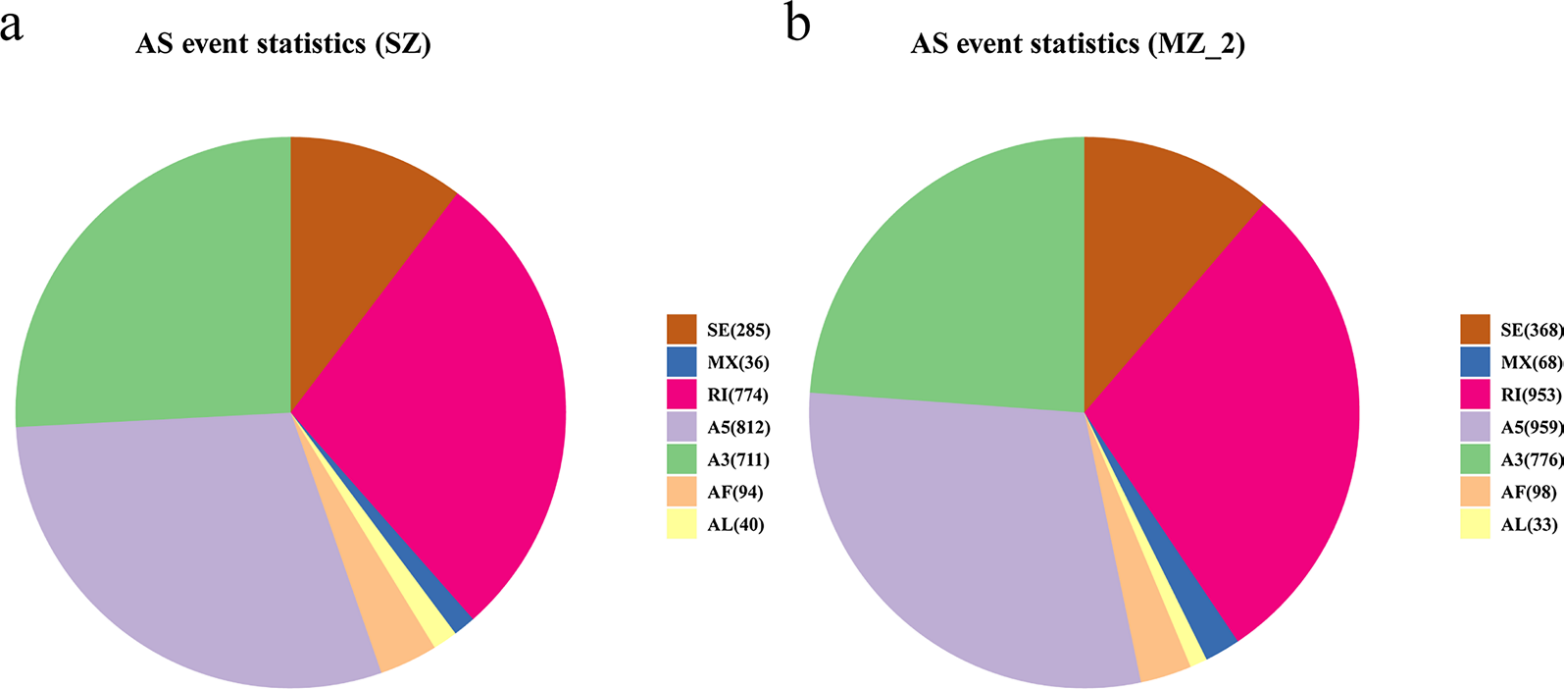
AS event analysis. **a**, **b** Total number of AS events detected in genes using SMRT in SZ (a) and MZ-2 (b). Abbreviations: A3, Alternative 3′ splice site; A5, alternative 5′ splice site; AF, alternative first exon; AL, alternative last exon; MX, mutually exclusive exon; RI, retained intron; SE, skipped exon

There were 2752 AS events that occurred specifically in 4254 genes of SZ, including 285 (10.36%) SE, 36 (1.31%) MX, 774 (28.12%) RI, 812 (29.50%) A5, 711 (25.84%) A3, 94 (3.42%) AF and 40 (1.45%) AL (Figs. 2a, 5a). Additionally, a total of 3255 AS events were detected in 4007 genes of MZ-2, including 368 (11.31%) SE, 68 (2.09%) MX, 953 (29.28%) RI, 959 (29.46%) A5, 776 (23.84%) A3, 98 (3.01%) AF and 33 (1.01%) AL (Figs. 2b, 5b). The majority of AS events in SZ and MZ-2 were A5 and RI, followed by A3, whereas MX and AL were least frequent.

### APA event analysis

**Fig. 6:**
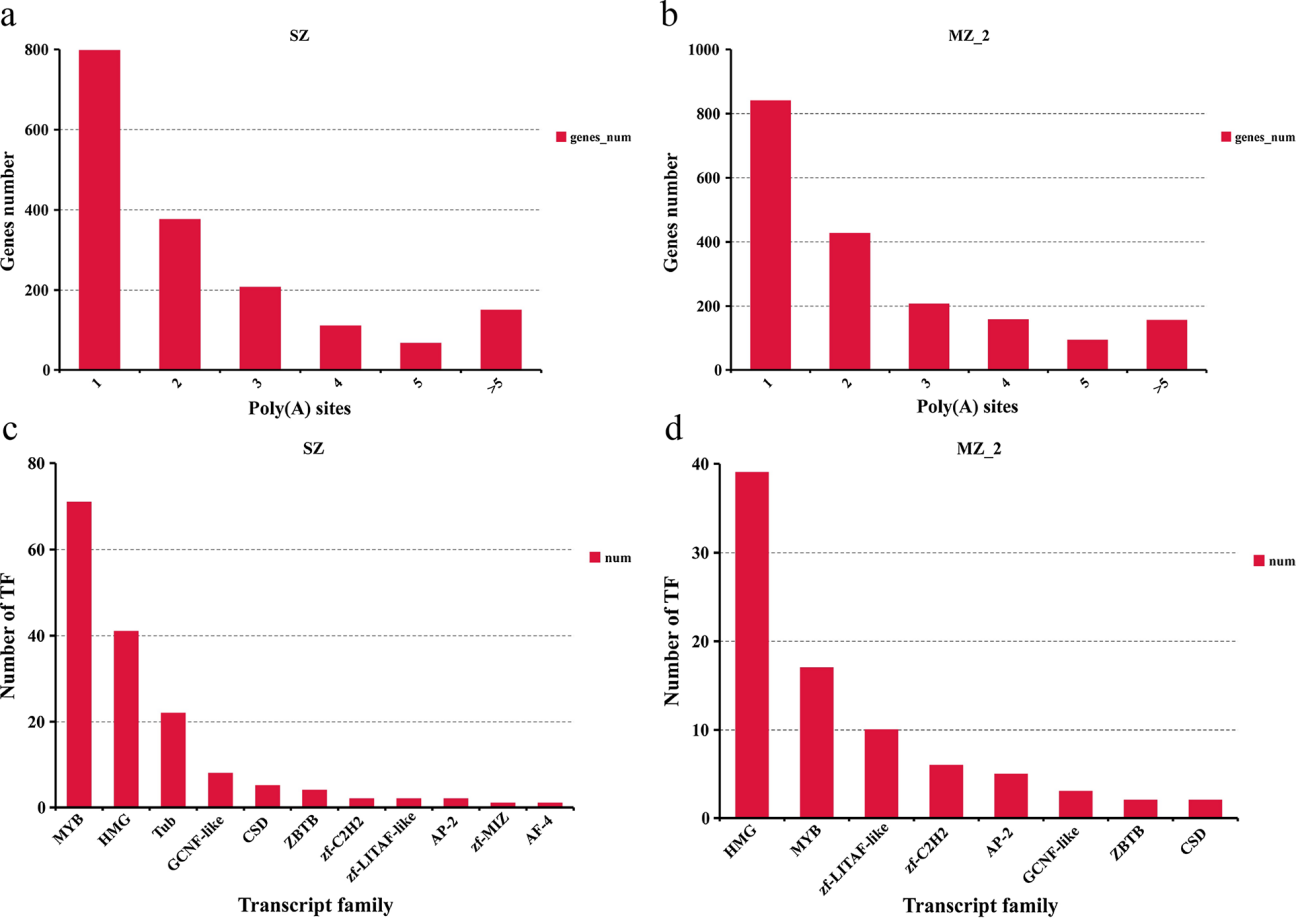
APA and TF analysis. **a**, **b** Distribution of the number of poly(A) sites per gene in SZ and MZ-2, respectively. **c**, **d** Number and family of putative TFs predicted by SMRT. Abbreviations: TF, transcript factor

Within the PacBio transcriptome, we detected 4415 APA events at 1705 genic loci in SZ. The APA events at genic loci were further compared with reference genes, which led to the detection of 797 genes with one poly(A) site, 376 genes with two poly(A) sites, 207 genes with three poly(A) sites, 109 genes with four poly(A) sites, 66 genes with five poly(A) sites and 150 genes with more than five poly(A) sites (Figs. 2a, 6a; Additional file 10: Table S6). The average number of poly(A) sites per gene was 2.59. We further detected 4629 APA events at 1874 genic loci in MZ-2, including 840 genes with one poly(A) site, 426 genes with two poly(A) sites, 206 genes with three poly(A) sites, 156 genes with four poly(A) sites, 92 genes with five poly(A) sites and 154 genes with more than five poly(A) sites (Figs. 2b, 6b; Additional file 10: Table S6). The average number of poly(A) sites per gene was 2.47.

### Identification of fusion transcripts, TFs and lncRNAs

Using the Illumina pair-end read validation approach, a total of 6607 (SZ, 4019; MZ-2, 2588) fusion transcripts were detected. All of the fusion events occurred inter-chromosomally. Our results revealed that all fusion events involved two or more genes (Fig. 2a, b; Additional file 11: Table S7). To further validate the fusion transcripts, 16 candidates were selected for RT-PCR analysis (Additional file 12: Figure S5), and results showed that the accuracy reached 87.5%. The PCR products were also confirmed using Sanger sequencing, and the results confirmed the authenticity of the chimeric transcripts.

A total of 159 putative TF members from 11 gene families and 84 putative TF members from eight gene families were identified in SZ and MZ-2, respectively. In SZ, 71, 41, 22, 8, 5, 4, 2, 2, 2, 1 and 1 putative TF member were identified from the MYB, HMG, Tub, GCNF-like, CSD, ZBTB, zf-C2H2, zf-LITAF-like, AP-2, zf-MIZ and AF-4 TF gene families, respectively (Fig. 6c). In MZ-2, 39, 17, 10, 6, 5, 3, 2 and 2 putative TF members were detected from the HMG, MYB, zf-LITAF-like, zf-C2H2, AP-2, GCNF-like, ZBTB and CSD TF gene families, respectively (Fig. 6d).

**Fig. 7:**
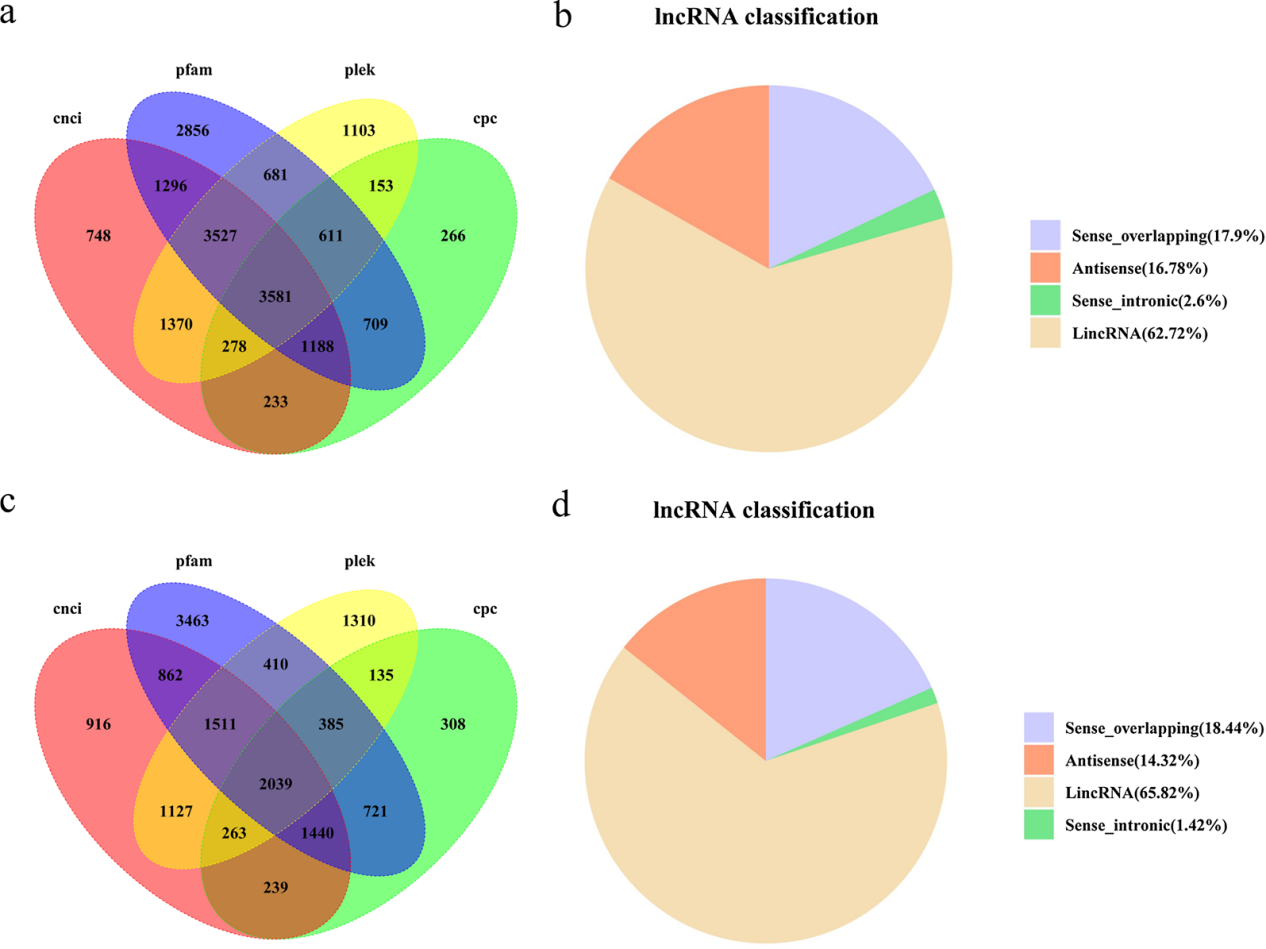
Identification of lncRNAs.** a**,** c** Venn diagram of lncRNAs predicted in SZ (a) and MZ-2 (c), using the CPC, CNCI, PLEK and Pfam methods. **b**, **d** Proportions of the four types of lncRNAs in SZ (b) and MZ-2 (d). Abbreviations: cnci, Coding-Non-Coding Index; plek, predictor of long non-coding RNAs and mRNAs based on an improved k-mer scheme; cpc, Coding Potential Calculator

We identified 3581 lncRNAs in SZ using four methods (CPC, 7019; CNCI, 12,221; PLEK, 11,304; Pfam, 14,448), 2130 (59.48%) of which were single exons (Figs. 2a, 7a; Additional file 13: Table S8). We classified these into the following four groups: lincRNA (2246, 62.72%), sense_intronic (93, 2.60%), antisense (601, 16.78%) and Sense_overlapping (641, 17.90%) (Fig. 7b). Similarly, a total of 2039 lncRNAs were detected in MZ-2 using the same four methods (CPC, 5530; CNCI, 8397; PLEK, 7180; Pfam, 10,830), 1130 (55.42%) of which were single exons (Figs. 2b, 7c; Additional file 13: Table S8). We classified these into the following four groups: lincRNA (1342, 65.82%), sense_intronic (29, 1.42%), antisense (292, 14.32%) and sense_overlapping (376, 18.44%) (Fig. 7d).

### Comparative analysis of transcript structures between SZ and MZ-2

Based on SMRT sequencing, we conducted a comparative analysis of SZ and MZ-2 transcript structures. Results showed that 3710 consensus isoforms were simultaneously mapped to the reference genomes of SZ and MZ-2, 19,330 consensus isoforms were expressed specifically in SZ and 18,213 consensus isoforms were expressed only in MZ-2. In addition, 815 AS events occurred simultaneously in SZ and MZ-2, 1937 AS events occurred specifically in SZ and 2440 AS events occurred only in MZ-2. Furthermore, a total of 1139 genes with APA sites, 20 putative TF members and 352 lncRNAs were identified in both SZ and MZ-2 (Additional file 14: Table S9).

## Discussion

Until now, Illumina sequences of *E. necatrix* MZ-2, third-generation merozoites and gametocytes have been published [26, 42], but SMRT sequences of *E. necatrix* had not been fully explored, and the full-length mRNA sequences, AS transcripts, APA sites and fusion transcripts of *E. necatrix* SZ and MZ-2 were unknown. Full-length transcripts can greatly improve genome annotation information and provide deep insights into the transcriptional landscape [43]. In the present study, we combined short Illumina sequences and high-accuracy reads for correction of SMRT sequences to generate an isoform dataset with high confidence and provided new insights into full-length sequences, gene structure, TFs and lncRNAs. A total of 222,019 and 192,045 corrected polished consensus reads were obtained from SZ and MZ-2, respectively. In addition, 23,040 (SZ) and 21,923 (MZ-2) consensus isoforms and 243 (SZ, 159; MZ-2, 84) putative TFs from 19 (SZ, 11; MZ-2, 8) gene families were detected. These new findings provide important information for improving *E. necatrix* genome annotation and fully characterizing the *E. necatrix* transcriptome.

AS events are a vital post-transcriptional regulatory mechanism that contributes to transcriptome and proteome complexity and diversity [44]. AS events occur mainly in plants during many developmental processes and in response to environmental cues [43, 45]. In *Eimeria nieschulzi*, the *gam56* and *gam82* genes encode proteins with smaller masses than avian *Eimeria* GAM proteins owing to AS [46]. In *Toxoplasma gondii*, ROP17 decreases AS events in host cells via altered expression of genes involved in the AS pathway to promote its own colonization and survival [47]. AS is an integral, stage-specific phenomenon in protists and a regulator of cellular differentiation that arose early in eukaryotic evolution and was required for sex-specific differentiation of gametocytes [48]. However, few studies have focused on AS events in *E. necatrix*. In the present study, we found that the majority of AS events in SZ and MZ-2 were alternative 5’ and retained introns, followed by 3’ splice sites. Previous reports showed that intron retention was the most frequently occurring type of AS event in plants [49–51]. Similarly, our findings revealed that intron retention events were most common in *E. necatrix*. We also found that 815 AS events occurred simultaneously in SZ and MZ-2, whereas 1937 AS events occurred specifically in SZ and 2440 AS events occurred only in MZ-2, indicating potential correlations between AS events and stage conversions.

APA increases transcript diversity and complexity by regulating RNA transportation, localization, stability and translation [32, 52, 53]. APA can regulate gene expression during plant biological processes including growth, development and the stress response [54, 55]. RNA 3’ end cleavage and polyadenylation sites occur in human genes and contribute to human diseases, including cancer and hematological, immunological and neurological diseases [56]. In *Sarcocystis neurona*, APA is widespread and has the potential to impact growth and development [57]. In *Trypanosoma brucei*, APA involving mitochondrial RNA polymerase gene mRNA produces two mature transcripts that show stage-specific differences in abundance during the life cycle [58]. In the present study, PacBio sequencing identified 1705 and 1874 genes with APA sites in SZ and MZ-2 of *E*. *necatrix*, respectively. These results greatly contribute to our understanding of the role of APA in *E. necatrix* gene regulation. Prediction of AS and APA sites also plays a crucial role in understanding stage differentiation in *Eimeria*. Our results showed that Iso-Seq has great potential for detecting AS and APA events in parasites.

Fusion transcripts are two or more separate genes joined into one transcript [33]. The generation of fusion transcripts involves the splicing machinery, indicating either trans-splicing of distinct genes or splicing of chimeric genes formed by somatic chromosomal rearrangements [59]. Gene fusion is a common feature in plants [34, 45, 60, 61], but only a few examples of gene fusion have been described in parasites. In *Eimeria*, the microgametocyte fusion protein EtHAP2 has been identified and is considered to be a novel vaccine candidate for interrupting parasite transmission [14]. In this work, we identified 4019 and 2588 fusion transcripts in SZ and MZ-2, respectively. The fusion events all occurred inter-chromosomally, which is consistent with the higher proportion of inter-chromosomal compared with intra-chromosomal fusions described in red clover and maize [34, 45]. These chimeric fusion events enhance the complexity of the *E. necatrix* transcriptome. To confirm the fusion transcripts, we randomly selected 16 candidates for RT-PCR analysis, 14 of which were validated by RT-PCR and Sanger sequencing. The findings concerning fusion transcripts could greatly improve draft versions of gene models in *E. necatrix*.

TFs are proteins that bind to DNA in a sequence-specific manner, and they play a crucial role in transcriptional regulation in eukaryotes. Previous studies reported that TFs coordinate many important biological processes, from cell cycle progression and physiological responses to cell differentiation and development [45]. The apicomplexan AP2 (ApiAP2) family of DNA-binding proteins is a major class of transcriptional regulators and has been extensively investigated as a potential regulator of differentiation [62, 63]. In *Plasmodium*, AP2-O is an AP2 family TF that directly regulates 10% of the parasite genome, is expressed during the mosquito midgut-invasion stage and is essential for stage-specific transcriptional regulation [64]. In *Eimeria*, 44–54 genes contain ApiAP2 domains, including 21 *Eimeria*-specific ApiAP2 groups, 22 groups shared by *Eimeria* and other coccidia and five pan-apicomplexan clusters [21]. Thirty-seven transcripts contain ApiAP2 domains, of which 12 are upregulated in gametocytes and seven are upregulated in third-generation merozoites in *E. necatrix* [42]. In the present study, we identified seven AP2 target transcripts, including two in SZ and five in MZ-2. The expression of AP2 family TFs throughout the life cycle implicates members of this family as major regulators of gene expression at all stages of *E. necatrix* development.

The MYB family of proteins, which was first characterized in the avian myeloblastosis virus, is highly conserved in eukaryotes, belongs to the tryptophan cluster family and regulates gene expression during differentiation and growth by binding to DNA [65]. In *Entamoeba histolytica*, MYB TFs may be involved in transcriptional regulation and participate in pathways related to virulence and the heat shock response [66]. In *Plasmodium*, PfMyb1 is essential for parasite growth, binding a number of promotors directly regulating key genes involved in cell cycle regulation and progression [67]. Here, we also detected MYB TFs in both SZ and MZ-2. Interestingly, MYB TFs were the most common protein family in *E. necatrix*. These results suggest that MYB TFs may be essential for parasite growth and may regulate expression of genes involved in stage differentiation.

lncRNAs are an emerging field that is rapidly evolving in the genome biology of specific species. However, what little is known about their function stems mostly from research on human cells and involves their role in transcriptional and epigenetic regulation [68–70]. Previous studies revealed that parasite-regulated lncRNAs were related to mRNA transcripts associated with the immune response [71]. In addition, hundreds of lincRNAs displaying evolutionary conservation, epigenetic marks of transcriptional activation, differential expression across different developmental stages and expression correlated with their protein-coding gene neighbors have been identified in *Schistosoma mansoni* [72]. In *P. falciparum*, a family of 22 telomere-associated lncRNAs was found to play an important role in telomere maintenance, virulence gene regulation and potentially other processes involving parasite chromosome end biology [73]. In *T. gondii*, non-coding RNA responses are likely to be major determinants of the ability of the host to resist infection and the ability of parasites to establish long-term latency [74]. Differentially expressed lncRNAs are thought to be involved in immune-related biological processes, nutritional absorption, biosynthesis and metabolism processes in *E. necatrix*-infected chickens [75]. In our research, we detected 3581 (SZ) and 2039 (MZ-2) lncRNAs as single-molecule transcripts. These lncRNAs may participate in regulation of gene expression and contribute to stage differentiation in *E. necatrix*. However, these expressed lncRNAs have not been well characterized and require further study.

Comparative analysis of transcripts between SZ and MZ-2 revealed some common consensus isoforms, but the majority of isoforms displayed stage-specific expression. Information on these stage-specific full-length transcripts enrich the *E. necatrix* database. Similarities and differences between SZ and MZ-2 were also found in gene structures such as AS, APA and TFs, which increase transcript diversity and functional complexity of genes. In addition to protein-encoding RNAs, non-coding RNAs are wide-spread in *E. necatrix*, a total of 3228 SZ-specific and 1687 MZ-2-specific lncRNAs were detected in this work. These stage-specific lncRNAs could be effectively applied as biomarkers or therapy targets in coccidiosis control programs.

## Conclusions

Based on SMRT RNA sequencing, we identified a large number of long-read isoforms in SZ and MZ-2 of *E. necatrix* and predicted that lncRNAs, AS and APA events may be involved in stage differentiation. Additionally, information on TFs provides a solid foundation for a better understanding of transcriptional regulation and fusion events will greatly improve draft versions of gene models in *E. necatrix*. RNA-sequencing analysis of gene structures will improve the understanding of the mechanisms governing the development of *Eimeria* parasite as well as aid in the development of novel strategies for coccidiosis control.

## Supplementary Information

**Additional file 1: Figure S1.** Purified MZ-2. MZ-2 samples purified from the chicken intestinal mucosal by Percoll density gradients method. Scale bars: 50 μm.

**Additional file 2: Figure S2.** Quality assessment of MZ-2 samples DNase-treated total RNA by agarose gel electrophoresis.

**Additional file 3: Figure S3.** Detection of parasite-specific large ribosomal RNA bands (28 S and 18 S) in MZ-2 using Agilent 2100.

**Additional file 4: Table S1.** Statistics of MZ-2 SMRT sequencing data.

**Additional file 5: Table S2.** Primers used for RT-PCR validation.

**Additional file 6: Figure S4.** Length distributions of PacBio SMRT sequencing.** a** Number and length distributions of subreads in MZ-2.** b** Number and length distributions of FLNC sequences in MZ-2.** c** Number and length distributions of consensus isoforms in MZ-2.

**Additional file 7: Table S3.** Statistics of MZ-2 Illumina sequencing data.

**Additional file 8: Table S4.** Distribution of transcript lengths before and after correction in MZ-2.

**Additional file 9: Table S5.** GMAP analysis of polished consensus isoforms to reference genome.

**Additional file 10: Table S6.** APA sites of genes detected by SMRT.

**Additional file 11: Table S7.** Information of fusion transcripts from Iso-Seq.

**Additional file 12: Figure S5.** Verification of 16 fusion transcripts by RT-PCR.

**Additional file 13: Table S8.** Exon number of lncRNAs from Iso-Seq.

**Additional file 14: Table S9.** Comparative analysis of transcripts structure between SZ and MZ-2.

## Data Availability

The PacBio SMRT reads and the Illumina SGS reads generated in this study have been submitted to the NCBI Sequence Read Archive (SRA; http://www.ncbi.nlm.nih.gov/sra) under accession number PRJNA730346 and PRJNA753889.

## Abbreviations

A3: Alternative 3′ splice sites
A5: Alternative 5′ splice sites
AF: Alternative first exons
AL: Alternative last exons
APA: Alternative polyadenylation
AS: Alternative splice
CCS: Circular consensus sequence
CNCI: Coding-Non-Coding Index
CPC: Coding Potential Calculator
FLNC: Full-length non-chimeric
GO: Gene Ontology database
Iso-Seq: Isoform sequencing
KEGG: Kyoto Encyclopedia of Genes and Genomes database
KOG: Eukaryotic Ortholog Groups
LincRNA: Long intergenic non-coding RNA
LncRNA: Long non-coding RNA
mRNA: messenger RNA
MX: Mutually exclusive exons
MZ-2: Second-generation merozoites
NR: NCBI non-redundant protein
NT: NCBI non-redundant nucleotide
Pfam: Protein families databas
PLEK: Predictor of long non-coding RNAs and mRNAs based on an improved k-mer scheme tool
RI: Retained introns
SE: Skipped exons
SMRT: Single-molecule real-time sequencing
SZ: Sporozoites
TF: Transcript factor
