# Supplementary figures and images for: Full-length transcriptome analysis and identification of transcript structures in Eimeria necatrix from different developmental stages by single-molecule real-time sequencing
Source: Parasit Vectors. 2021 Sep 27;14:502. doi: 10.1186/s13071-021-05015-7 (PMC8474931; doi:10.1186/s13071-021-05015-7)

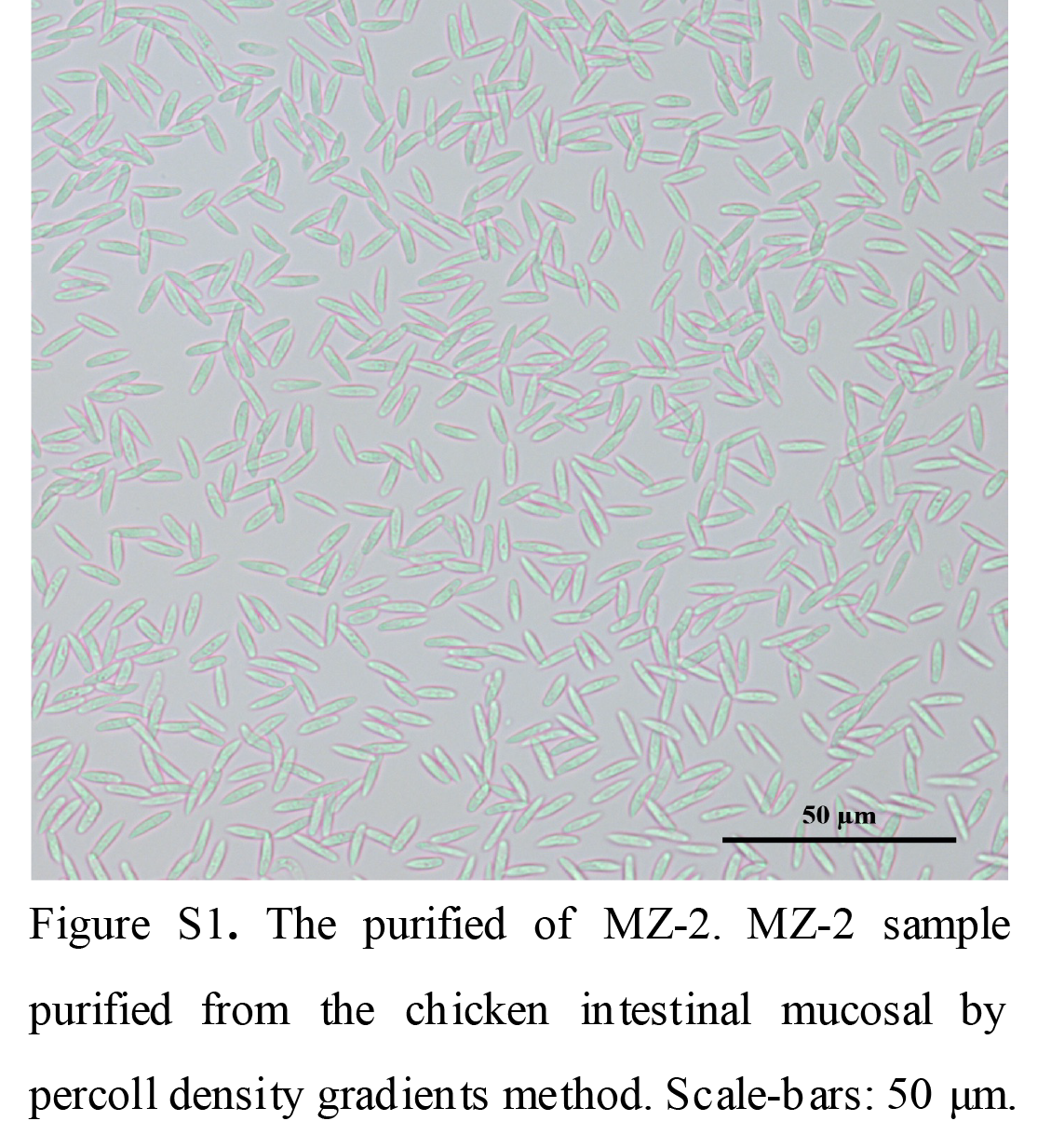

Supplement: Supplementary file 1 — Additional file 1: Figure S1. Purified MZ-2. MZ-2 samples purified from the chicken intestinal mucosal by Percoll density gradients method. Scale bars: 50 μm. [file 13071_2021_5015_MOESM1_ESM.tif]

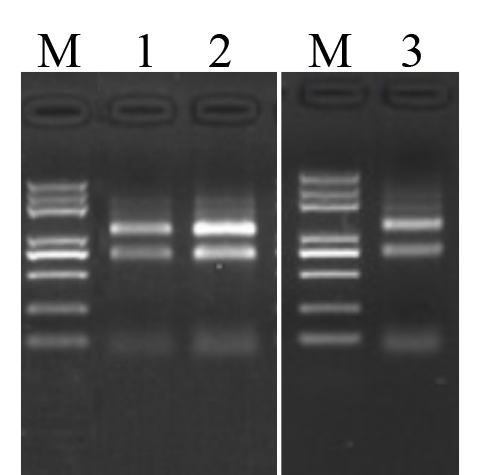

Supplement: Supplementary file 2 — Additional file 2: Figure S2. Quality assessment of MZ-2 samples DNase-treated total RNA by agarose gel electrophoresis. [file 13071_2021_5015_MOESM2_ESM.tif]

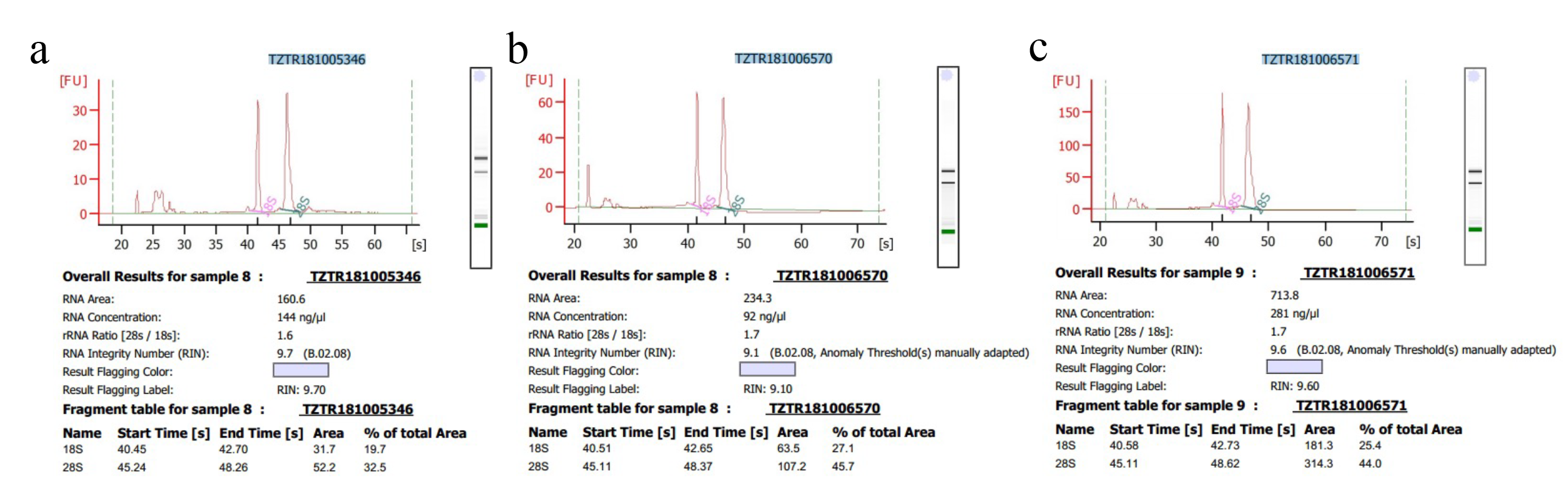

Supplement: Supplementary file 3 — Additional file 3: Figure S3. Detection of parasite-specific large ribosomal RNA bands (28 S and 18 S) in MZ-2 using Agilent 2100. [file 13071_2021_5015_MOESM3_ESM.tif]

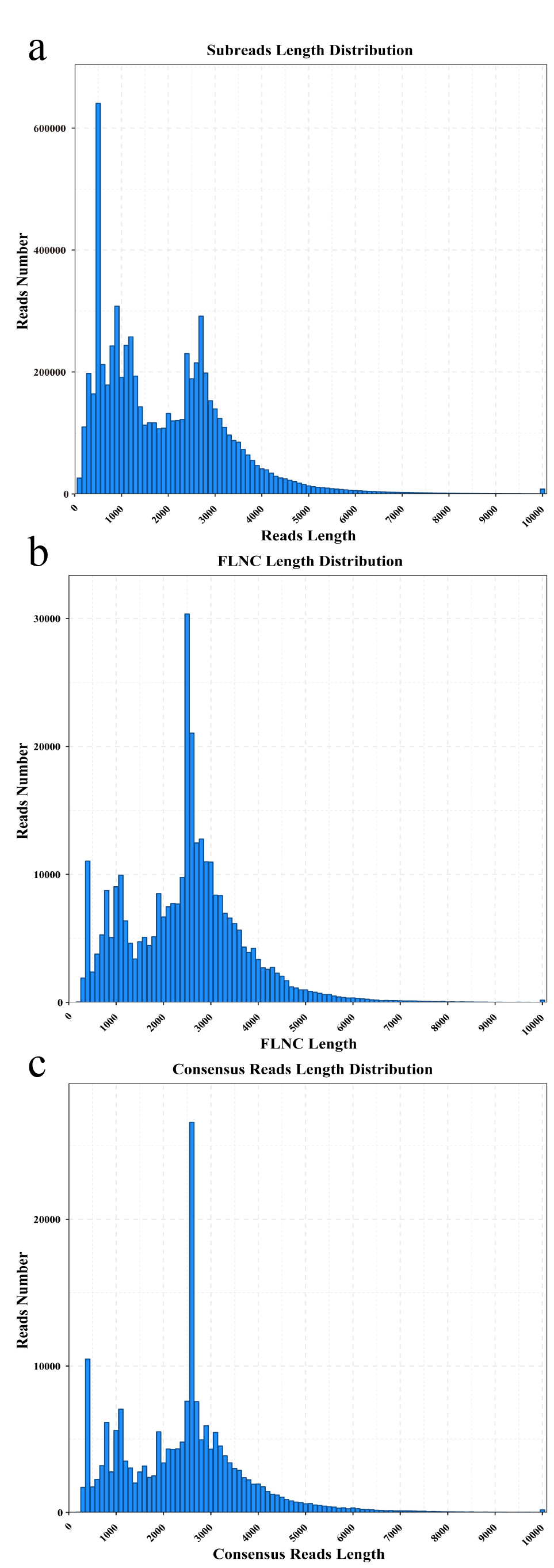

Supplement: Supplementary file 6 — Additional file 6: Figure S4. Length distributions of PacBio SMRT sequencing. a Number and length distributions of subreads in MZ-2. b Number and length distributions of FLNC sequences in MZ-2. c Number and length distributions of consensus isoforms in MZ-2. [file 13071_2021_5015_MOESM6_ESM.tif]

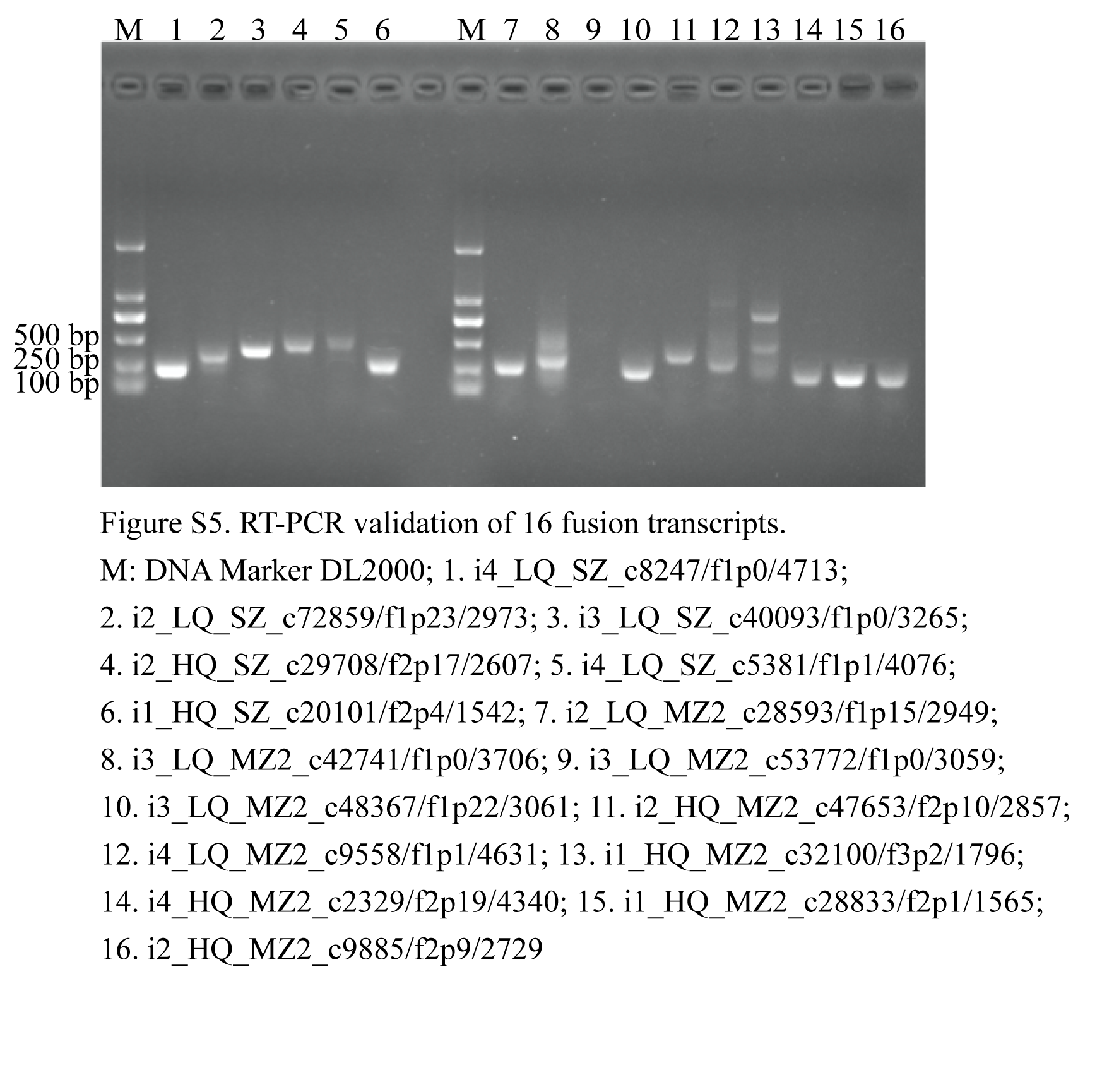

Supplement: Supplementary file 12 — Additional file 12: Figure S5. Verification of 16 fusion transcripts by RT-PCR. [file 13071_2021_5015_MOESM12_ESM.tif]
